# Epidemiological surveillance of bacterial pathogens in cultured European sea bass (*Dicentrarchus labrax*) along the Aegean coast of Türkiye

**DOI:** 10.1371/journal.pone.0362366

**Published:** 2026-11-06

**Authors:** Pınar Çelik, Vyron Georgalas, İhsan Çelik

**Affiliations:** Çanakkale Onsekiz Mart University, Faculty of Marine Sciences and Technology, Department of Aquaculture, Çanakkale, Türkiye; Tanta University Faculty of Agriculture, EGYPT

## Abstract

Intensive aquaculture of European sea bass (*Dicentrarchus labrax*) along the Aegean coast of Türkiye faces continuous production challenges from infectious diseases, yet comprehensive multi-year baseline data remain limited. This study presents a multi-year retrospective surveillance program (2020–2025) monitoring bacterial pathogens associated with 748 distinct cage-level disease outbreaks in fish across 19 discrete offshore commercial sea farms. To maintain industry confidentiality, diagnostic data from these separate facilities were fully aggregated and evaluated across three broad macro-regional surveillance zones. A total of 5,235 fish displaying clinical signs underwent diagnostic necropsy. Presumptive bacterial identification was conducted using phenotypic profiling through the API 20E system, and organ touch-imprint cytology. Six distinct bacterial species were isolated from positive clinical individuals, recovering a total of 2,490 isolates. Across the recorded outbreak events, etiological agents were ranked as primary drivers as follows: *Photobacterium damselae* subsp. *piscicida* (48.80%; *N* = 365), *Tenacibaculum maritimum* (35.83%; *N* = 268), *Vibrio (Listonella) anguillarum* (9.49%; *N* = 71), *V. harveyi* (3.74%; *N* = 28), *V. ordalii* (1.20%; *N* = 9), and *V. alginolyticus* (0.94%; *N* = 7). This hierarchy directly mirrored the overall bacterial isolation frequency, where *P. damselae* subsp. *piscicida* showed the highest relative distribution rate (46.18%; *n* = 1,150), followed by *T. maritimum* (34.10%; *n* = 849). Significantly lower isolation rates were recorded for *V.* (*Listonella*) *anguillarum* (11.41%; *n* = 284), *V. harveyi* (5.26%; *n* = 131), *V. ordalii* (1.77%; *n* = 44), and *V. alginolyticus* (1.29%; *n* = 32). Descriptive tracking of the purified laboratory isolates revealed localized variations in diagnostic recovery frequencies among the three sectors, while establishing a highly stable baseline dominated by *P. damselae* subsp. *piscicida* and *T. maritimum* across the Eastern Aegean Turkish mariculture network.

## Introduction

Aquaculture is one of the fastest-growing sectors in global animal food production [1,2]. This industry plays an increasingly critical role in meeting the nutritional needs of a growing global population with seafood, while providing employment and supporting economic growth [2–5]. In Europe alone, aquaculture production increased by 101% from 1999 to 2009 and by 69% from 2009 to 2019 [5]. This expansion is even higher in Asia [1]. This serves as a strong indicator of how rapidly the global aquaculture industry is expanding. European aquaculture accounts for 83% of production in the region as marine fish [5]. Türkiye is also among the leading countries in Europe for aquaculture [2,5]. In 2024, Türkiye produced a total of 165,550 tons of European sea bass (*Dicentrarchus labrax*) and 155,279 tons of gilthead sea bream (*Sparus aurata*), reflecting a steadily expanding production trajectory. Türkiye currently hosts 2,139 aquaculture facilities, of which 434 operate in marine environments, with the majority dedicated to European sea bass mariculture [6].

However, rapid industrial expansion introduces significant operational and managerial challenges, such as handling fluctuating seasonal temperatures, optimizing stocking densities and variable water quality parameters. These environmental stressors directly influence pathogen dynamics and virulence, increasing disease outbreaks and production costs [2,6]. In marine aquaculture, gross pathological examination remains a critical frontline tool for rapid disease diagnosis, allowing specialists to identify characteristic tissue lesions and implement immediate treatments before receiving laboratory confirmation [7,8]. While isolated disease cases have been reported in Türkiye [9–12], a comprehensive, long-term regional baseline of field pathology trends across multiple years has, to our knowledge, been lacking. To address this gap, this study presents a multi-year surveillance campaign (2020–2025) examining 5,235 symptomatic fish across 19 discrete offshore commercial sea farms. To preserve industry confidentiality, diagnostic data from these individual facilities were aggregated and evaluated across three broad macro-regional sectors along the Aegean coast. The primary aim of this surveillance campaign was to catalog regional bacterial profiles, describe their associated gross lesions, and document macro-regional descriptive frequency distributions in cage-reared sea bass along the Aegean coast.

## Materials and methods

### Ethics Statement and Site Access

This observational field surveillance study did not involve experimental manipulation, intentional infection, or physiological modification of live animals. Therefore, formal institutional animal care and use committee protocol review was not required under local veterinary diagnostic regulations. Per national legislation (Turkish Republic Ministry of Agriculture and Forestry, Regulation on the Protection and Welfare of Animals Used for Scientific Purposes, Official Gazette No. 28141 [13]), formal institutional animal ethics committee (IACUC/HADYEK) protocol review is strictly restricted to active experimental interventions on live animals. According to Article 2, paragraph 2(b) of this national decree, non-experimental clinical veterinary medicine practices are explicitly excluded from the scope of mandatory institutional ethics committee coding.

Field sampling was conducted utilizing an active, event-driven convenience sampling approach, encompassing commercial mariculture operations along the Turkish eastern Aegean Sea coastline that formally requested diagnostic assistance during active field mortality events. To track infection dynamics without compromising commercial confidentiality, a standardized case definition was established as an official trigger of field mobilization and targeted sample collection. A clinical outbreak event was registered if daily fish mortality in a specific cage—excluding losses attributable to environmental anomalies or routine farm operations—exceeded a predefined operational threshold of 2% of the actual cage population for three consecutive days. All sample collections and post-mortem investigations were performed during these triggered diagnostic visits with the explicit permission and cooperation of the facility management. To cleanly distinguish between outbreaks within the same cage, a strict 15-day rule was applied. A new outbreak was registered if a different pathogen was identified as the primary mortality driver within a 15-day window, or if the same pathogen re-emerged as the primary mortality driver more than 15 days after the initial sampling date. Conversely, concurrent tracking of the same pathogen as the primary mortality driver within a 15-day block was recorded as a single continuous outbreak. While sampling events occurred across 19 discrete offshore commercial aquaculture facilities operating continuously throughout the multi-year study window (5 facilities in the Northern zone, 6 facilities in the Central zone, and 8 facilities in the Southern zone), all diagnostic data were aggregated across three broad macro-regional sectors—the Northern, Central, and Southern Aegean Surveillance Zones—to strictly protect corporate anonymity and maintain stakeholder confidentiality (Fig 1). Constraints regarding farm-, cage-, and outbreak-level data clustering are addressed as a limitation of this event-driven convenience sampling approach.

**Fig 1:**
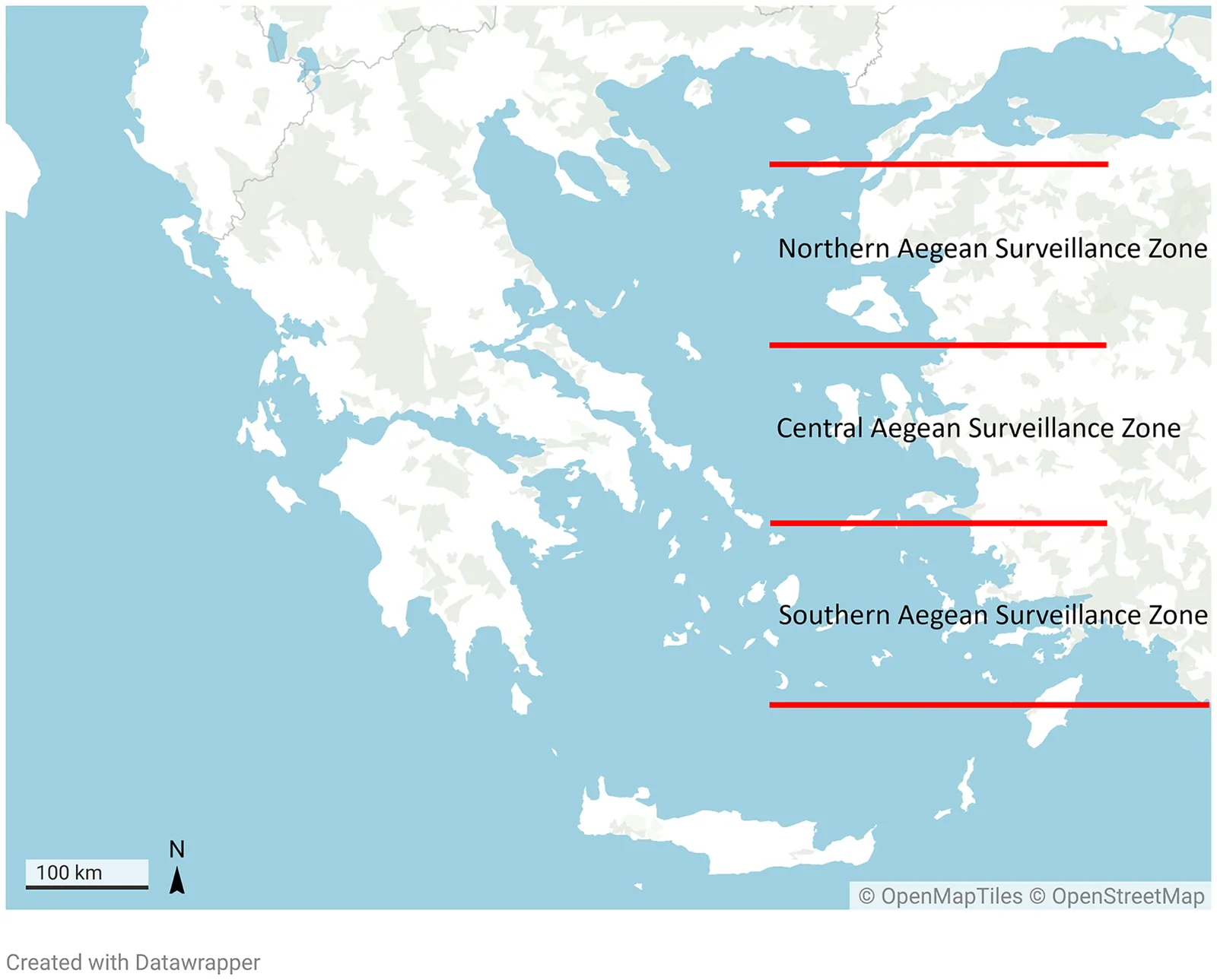
Delineation of macro-regional epidemiological surveillance zones along the Aegean coast of Türkiye. The three broad spatial sectors evaluated over the multi-year surveillance campaign are delineated by macro-regional horizontal boundaries: the Northern Aegean Surveillance Zone, Central Aegean Surveillance Zone, and Southern Aegean Surveillance Zone. The base map layers and regional coastline configurations are generated using open-source, CC BY 4.0-compliant map layouts via Datawrapper (datawrapper.de) utilizing geographic data boundaries from OpenMapTiles and OpenStreetMap contributors.

Because field mobilization was strictly contingent upon crossing the mortality threshold, the resulting surveillance pool was inherently pre-selected for active, advanced disease states rather than baseline population monitoring. This targeted operational framework was explicitly structured to optimize the capture of clinically positive individuals and maximize pathogen isolation yields from the collected samples.

### Sample collection and euthanasia protocols

Field surveillance was conducted across the 19 discrete commercial farms distributed within the three designated macro-regional aquaculture zones from January 2020 to October 2025. Prior to sample collection, each cage was macroscopically inspected from the surface to evaluate and record active swimming behaviors, schooling patterns, and explicit clinical abnormalities. Concurrently, historical management logs for each targeted cage—including recent handling events, recent mortality pattern, stocking density, and prior therapeutic interventions—were systematically documented. Following this initial behavioral appraisal during the natural disease outbreak, representative samples of moribund or freshly dead fish (2.5–2,500 g) were collected. Freshly dead fish were immediately placed in individual plastic bags and stored on crushed ice.

Moribund fish were euthanized using a size-dependent protocol following AVMA guidelines [14]. Moribund juveniles under 200 g were euthanized via rapid hypothermia by immersion in an ice-water slurry (<2°C) before being individually bagged. Larger individuals over 200 g were first immersed in a lethal overdose of clove oil (*Syzygium aromaticum* extract; 50–100 mg/L). Fish remained in the anesthetic solution for 10 minutes past opercular movement cessation to ensure permanent brain death, then they were transferred to the ice-water slurry (<2°C) before being individually bagged. All samples were transported on crushed ice to the laboratory within hours for examination.

In the laboratory, all collected fish underwent complete gross pathological and systematic post-mortem examinations following standardized veterinary diagnostic protocols [15,16]. Prior to internal organ sampling, the external skin surface of each euthanized fish was thoroughly disinfected with 70% ethanol to eliminate superficial environmental contaminants, followed by a ventral midline incision to expose the visceral mass. For rapid cytological verification of bacterial pathomorphology directly from active field cases, fresh wet mounts were prepared from peripheral blood for immediate microscopic evaluation, while organ touch imprints from the spleen, liver, and brain were prepared on clean glass slides, air-dried, and heat-fixed. These tissue imprints were systematically evaluated using a modified Gram-Color staining kit (REF 1.01603.0001, Merck) under oil immersion at 1000 × magnification. Following primary internal exposure, a systematic gross pathological evaluation was conducted on all visceral organs. In rare clinical cases where individuals exhibited severe exophthalmia, eye fluid was additionally aseptically withdrawn following localized surface sterilization of the ocular surface.

To maximize diagnostic efficiency and eliminate redundant phenotypic testing of identical clonal presentations within a single cage outbreak event, full primary isolation was executed on a representative sub-sample of 2–7 highly symptomatic fish per primary disease event. These target specimens were selected adaptively based on initial high cytological bacterial loads and optimal biometric size to facilitate sterile handling. To maintain standard aseptic isolation criteria for kidney culturing, the structural integrity of the swimbladder was strictly verified, with specimens presenting a ruptured or damaged swimbladder being excluded from isolation attempts to prevent gastrointestinal cross-contamination. If bacterial presence was cytologically confirmed in the blood or spleen and the swimbladder was intact, all internal visceral organs were carefully excised to expose the intact swimbladder lining, and the remaining peritoneal cavity was thoroughly disinfected with 70% ethanol. A sterile scalpel blade was then heated and used to slice open the ventral wall of the swimbladder, which served as a sterile anatomical barrier protecting the underlying tissue. Finally, sterile loops were passed through this incision to harvest deep tissue fragments directly from the kidneys, which were immediately inoculated onto Brain Heart Infusion Agar (BHIA, Oxoid), Trypticase Soy Agar (TSA, Oxoid), Thiosulfate Citrate Bile Salts Sucrose (TCBS, Oxoid) agar or Marine Salt Agar with Blood (MSA-B) prepared according to standard protocols [7]. Alternatively, for primary culture, blood was drawn aseptically from the caudal vein or the heart atrium and used for plate inoculation. Plates were incubated at 25°C for 24–72 h to monitor colony morphology. For the isolation and culture of *Tenacibaculum maritimum*, Marine agar 2216 (DIFCO) was utilized via direct inoculation of superficial cutaneous lesions prior to alcohol disinfection. The diagnostic tracking workflow systematically distinguished among individual fish examined, tissue samples selected for laboratory processing, and final primary culture outcomes. Primary tissue cultivation attempts were categorized as unsuccessful and discarded from downstream phenotypic characterization based on three strict quality-control criteria: (1) an absolute absence of colonial development (no growth) after the full incubation timeline, (2) superficial plate contamination characterized by rapid saprophytic overgrowth developing entirely outside the primary streak lines, or (3) internal agar matrix contamination embedded within the commercial media that became visible only after exposure to the incubator environment. These compromised or unviable plates were systematically recorded as negative, autoclaved at 121°C for 20 minutes for biological neutralization, and routed to standard biohazard waste disposal.

Duplicate touch imprints of nodular spleen and kidney tissues were stained using a modified Acid-Fast staining kit (REF 1.16450.0001, Merck) to screen for acid-fast bacilli within the nodular structures. To ensure diagnostic validity, batch-specific *Mycobacterium* spp. positive control slides were processed concurrently with all field smears. This targeted screening was restricted to individual fish with gross nodular visceral lesions to evaluate the presence of acid-fast bacilli.

For isolated colonies streaked on solid growth media, baseline motility, Gram-staining, catalase production, and cytochrome oxidase activities were verified following standard identification keys [7,8,17,18]. Susceptibility to the vibriostatic agent O129 (2,4-diamino-6,7-diisopropylpteridine) was systematically evaluated using 10 µg (DD14) and 150 µg (DD15) diagnostic discs (Oxoid) on Tryptic Soy Agar (TSA) plates supplemented with 1.5% NaCl to differentiate *Vibrio* species from phenotypically similar, resistant marine rods. For *T. maritimum* isolates, the recorded non-motile profile refers strictly to the absence of active flagellar swimming motility in liquid suspension, distinguishing it from surface-associated gliding motility characteristic of the genus.

Primary colonies used for strip inoculation were standardized via cultivation on standard TSA plates for 48 h, with the strict exception of *T. maritimum* which were harvested directly from Marine agar 2216. Presumptive species-level identification was performed using the API 20E system (bioMérieux S.A.) [7,8,17,18] utilizing a 0.9% NaCl inoculum and following strip incubation at 25°C for 48 h.

### Data analysis

All epidemiological datasets and baseline tracking arrays were structurally cataloged, processed, and descriptively evaluated using Microsoft Excel (v16.0, Microsoft Corporation). To describe regional pathogen dynamics, baseline etiological frequencies were compiled and evaluated hierarchically across both nested data layers: the outbreak event level (utilizing the 748 distinct primary cage outbreaks) and the individual laboratory isolate level (utilizing the 2,490 purified bacterial isolates). Cross-classified percentage differences (Δ) were calculated between pathogen pairs across both analytical levels to provide a transparent overview of etiological dominance rankings. Because granular production facility registries, specific cage tracking logs, and precise temporal identifiers are strictly restricted due to contractually binding commercial anonymity agreements, formal inferential population-scale modeling, random-effects evaluations, and multi-level spatial clustering adjustments were contractually unfeasible. Consequently, all data layer allocations are strictly restricted to the descriptive baseline context of this event-driven convenience surveillance framework, mapping direct percentage frequencies and raw event distributions without inferential statistical overlays.

## Results

### Epidemiological denominators and surveillance demographics

During field surveillance, a total of 5,235 fish were macroscopically and cytologically examined during the 748 active outbreak events (Table 1). Outbreak events were categorized based on the primary systemic pathogen driving cage mortality. Characteristic pathomorphological signs of bacterial disease were clinically and cytologically confirmed in 5,025 fish (95.99%), while 210 fish (4.01%) were determined to be negative for the investigated pathogens. Clinical co-infections were identified in 142 fish (2.71%) based on concurrent gross pathological signs and cytological verification. Co-infections consistently involved exactly two bacterial pathogens per fish, strictly partitioned by anatomical site: a presumptive *Tenacibaculum maritimum* observed cytologically on external cutaneous lesions, paired with a single systemic pathogen driving mortality. Because full bacterial isolation was prioritized for the primary systemic kidney infections to track main mortality drivers, these cutaneous tenacibaculosis presentations were noted clinically but were not always subjected to downstream cultural isolation.

**Table 1:** Macro-regional baseline epidemiological distribution, primary etiological outbreak classification, and subsequent fish-level bacterial isolation frequencies recovered from cultured European sea bass (*Dicentrarchus labrax*) along the Turkish Aegean coast (2020–2025). Bacterial species columns under the population-scale group (*N* = 748 outbreak events) and the laboratory culture group (*n* = 2,490 purified isolates) are organized concurrently from left to right in descending order of overall recovery frequency. Note: Mixed clinical co-infections (*n* = 142 fish) are partitioned strictly by anatomical site (external cutaneous vs. internal systemic) and allocated under their primary systemic driver column to prevent cross-level double-counting. Pathogen-specific isolate columns reflect individual fish-level recoveries following an adaptive sub-sampling protocol of 2 to 7 highly symptomatic fish per active cage crisis.

Sampling metrics					Primary Etiological Drivers per Outbreak Event						Bacterial isolation metrics									
Surveillance Zone	Outbreaks recorded	Fish examined	Total positive fish	Fish with co-infection	*Photobacterium damselae* subsp. p*iscicida*	*Tenacibaculum maritimum*	*Vibrio (Listonella) anguillarum*	*Vibrio harveyi*	*Vibrio ordali*i	*Vibrio alginolyticus*	Bacterial isolation attempts	Sub-sampling Ratio (%)	Bacterial isolates obtained	Bacterial isolation success rate (%)	*Photobacterium damselae* subsp. p*iscicida*	*Tenacibaculum maritimum*	*Vibrio (Listonella) anguillarum*	*Vibrio harveyi*	*Vibrio ordali*i	*Vibrio alginolyticus*
Northern Aegean	253	1766	1695	55	122	87	30	8	4	2	1130	63.99	1064	94.16	483	359	140	54	17	11
Central Aegean	204	1432	1350	32	100	62	28	10	2	2	574	40.08	541	94.25	262	190	61	15	9	4
Southern Aegean	291	2037	1980	55	143	119	13	10	3	3	914	44.87	885	96.83	405	300	83	62	18	17
Total	748	5235	5025	142	365	268	71	28	9	7	2618	50.01	2490	95.11	1150	849	284	131	44	32

This diagnostic strategy yielded a baseline collection of 2,490 total purified primary bacterial isolates across the multi-year surveillance period (Table 1). The event-driven convenience sampling framework resulted in variable regional sub-sampling ratios, ranging from 40.08% in the Central Aegean to 63.99% in the Northern Aegean zone, yielding a collective study-wide sub-sampling ratio of 50.01% across the collective pool of 5,235 examined fish. Diagnostic processing demonstrated high technical efficiency, achieving regional primary isolation success rates between 94.16% (Northern Aegean) and 96.83% (Southern Aegean), resulting in a cumulative study success rate of 95.11%. The remaining 128 isolation attempts were unsuccessful and were thus excluded from further analysis. Consequently, all spatial and species-specific distribution profiles reported throughout this study correspond directly to their relative frequencies calculated within this verified primary isolate pool (*n* = 2,490).

An evaluation of the nested data hierarchy reveals highly distinct pathogen-specific distribution dynamics across the coastline. While the primary etiological drivers at the population scale track these mortality outbreaks —confirming septicemic pasteurellosis as the single most frequent driver of cage-level mortality crises (48.80%; 365 out of 748 outbreaks)—the individual isolate-level counts provide an accurate reflection of total laboratory recovery yields. Crucially, the sub-sampling yields remained highly disciplined and consistent across both dominant agents, averaging 3.15 isolates per *Photobacterium* outbreak and 3.17 isolates per *Tenacibaculum* outbreak, aligning perfectly with our adaptive 2-to-7 fish sub-sampling protocol. The recorded data report both the outbreak counts and the bacterial isolation frequencies documented along the coastline within the examined symptomatic fish.

### Bacterial Isolation and Phenotypic Profiling

The most common bacterial isolates (in descending frequency), were that of *Photobacterium damselae* subsp. *piscicida*, *Tenacibaculum maritimum*, *Vibrio* (Listonella) *anguillarum*, *V. harveyi*, *V. ordalii* and *V. alginolyticus* (see Table 1 for their geographical and surveillance zone distribution). Phenotypic and biochemical profiles for each species remained highly consistent across sampling locations and seasonal years (Table 2). The sole difference was observed in Citrate utilization (CIT) assays which demonstrated variable results within isolates of both *P. damselae* subsp. *piscicida* and *V.* (*Listonella) anguillarum*.

**Table 2:** Phenotypic and biochemical characteristics of the bacterial pathogens isolated from European sea bass (Dicentrarchus labrax) along the Turkish Aegean coast. (+) = positive reaction; (-) = negative reaction; V = variable reaction within regional field strains.

	Bacteria isolate					
Test	*Photobacterium damselae* subsp. p*iscicida*	*Tenacibaculum maritimum*	Vibrio (*Listonella) anguillarum*	*Vibrio harveyi*	*Vibrio ordalii*	*Vibrio alginolyticus*
**β-galactosidase (ONPG)**	**–**	**–**	**+**	**–**	**–**	**–**
**Arginine Dihydrolase (ADH)**	**+**	**–**	**+**	**–**	**–**	**–**
**Lysine Decarboxylase (LDC)**	**–**	**–**	**–**	**+**	**–**	**+**
**Ornithine Decarboxylase (ODC)**	**–**	**–**	**–**	**+**	**–**	**+**
**Citrate utilization (CIT)**	**V**	**–**	**V**	**+**	**–**	**–**
**H**_**2**_ **S production (H**_**2**_ **S)**	**–**	**+**	**–**	**–**	**–**	**–**
**Urease (URE)**	**–**	**–**	**–**	**–**	**–**	**–**
**Tryptophane Deaminase (TDA)**	**–**	**–**	**–**	**–**	**–**	**–**
**Indole production (IND)**	**–**	**–**	**+**	**+**	**–**	**+**
**Acetoin production (VP)**	**+**	**+**	**+**	**–**	**–**	**+**
**Gelatinase (GEL)**	**–**	**–**	**+**	**+**	**–**	**+**
**Glucose (GLU)**	**+**	**–**	**+**	**+**	**+**	**+**
**Mannitol (MAN)**	**–**	**–**	**+**	**+**	**–**	**+**
**Inositol (INO)**	**–**	**–**	**–**	**–**	**+**	**–**
**Sorbitol (SOR)**	**–**	**–**	**+**	**–**	**+**	**–**
**Rhamnose (RHA)**	**–**	**–**	**–**	**–**	**+**	**–**
**Sucrose (SAC)**	**–**	**–**	**+**	**+**	**–**	**+**
**Melibiose (MEL)**	**–**	**–**	**–**	**–**	**–**	**–**
**Amygdalin (AMY)**	**–**	**–**	**–**	**+**	**–**	**–**
**Arabinose (ARA)**	**–**	**–**	**–**	**–**	**+**	**–**
**Oxidase (OX)**	**+**	**+**	**+**	**+**	**+**	**+**
**NO**_**2**_ **production**	**–**	**+**	**+**	**+**	**+**	**+**
**Reduction to N**_**2**_ **gas**	**–**	**–**	**+**	**–**	**–**	**–**
**Motility (MOB)**	**–**	**–**	**+**	**+**	**+**	**+**
**Gram-negative**	**+**	**+**	**+**	**+**	**+**	**+**

The macroscopically observed gross clinical signs are reported in Table 3. Representative macro-pathological transformations, organ touch imprints, and wet-mount cytological diagnostic configurations are structurally detailed across these disease profiles (Fig 2).

**Table 3:** Comparative gross clinical signs in infected European sea bass. *Photobacterium damselae* subsp. p*iscicida, Tenacibaculum maritimum*, *Vibrio* (*Listonella) anguillarum, Vibrio harveyi*, *Vibrio ordalii*, *Vibrio alginolyticus*. (+) indicates presence of clinical sign; (-) indicates absence; (V) indicates variation in presentation.

		Bacteria isolate				
Gross signs	*Photobacterium damselae* subsp. p*iscicida*	*Tenacibaculum maritimum*	Vibrio (*Listonella) anguillarum*	*Vibrio harveyi*	*Vibrio ordalii*	*Vibrio alginolyticus*
**Lethargy**	**+**	**V**	**+**	**+**	**+**	**+**
**Anorexia**	**+**	**V**	**+**	**+**	**+**	**+**
**Dark body coloration**	**+**	**–**	**+**	**+**	**+**	**+**
**Ulcers on skin**	**–**	**+**	**+**	**+**	**–**	**+**
**Skin lesions**	**–**	**+**	**+**	**+**	**+**	**+**
**Pale gills**	**–**	**+**	**+**	**+**	**+**	**+**
**Abnormal swimming behavior**	**+**	**–**	**+**	**+**	**–**	**–**

**Fig 2:**
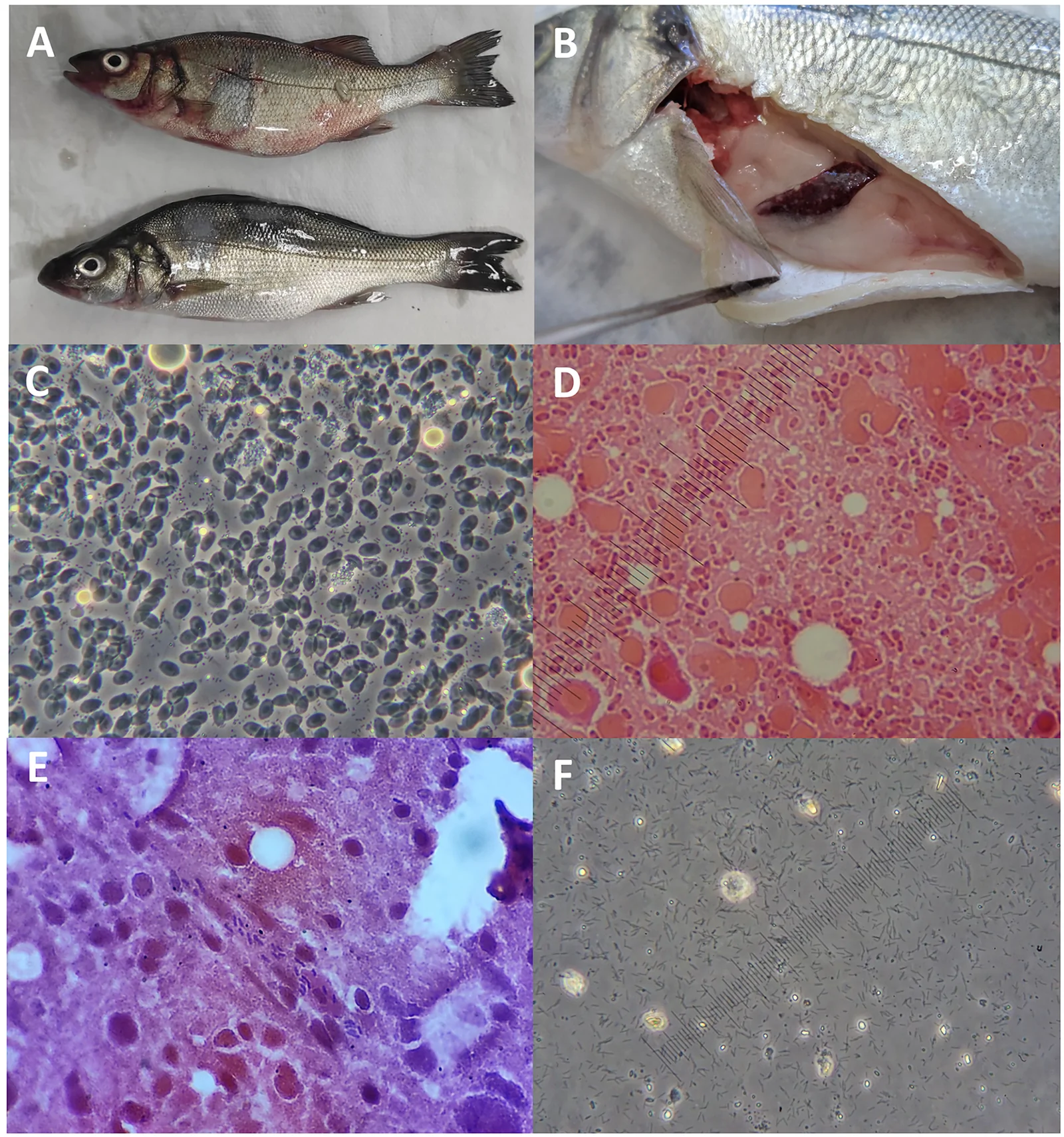
Pathomorphological, cytological, and diagnostic imprint findings in cultured European sea bass (*Dicentrarchus labrax*) naturally infected with marine bacterial pathogens. (A) Macroscopic clinical signs of diseased adult sea bass individuals displaying variable pathogenic manifestations: a highly symptomatic individual (top) exhibiting concurrent characteristic clinical signs, including extensive petechial hemorrhages on the ventral abdominal wall and severe opercular congestion typical of a suspected systemic *Vibrio* bacteremia, alongside extensive body lesions characterized by focal scale loss and erosion of the caudal fin margins consistent with tenacibaculosis later confirmed microscopically; contrasted against an individual (bottom) displaying localized cutaneous tenacibaculosis characterized by matching focal body lesions, skin ulcerations with scale loss, and fin margin erosions without systemic septicemic features. (B) Severe splenomegaly in an infected juvenile fish, characterized by prominent, widespread whitish necrotic nodules throughout the splenic parenchyma, later presumptively identified as *Photobacterium damselae* subsp. *piscicida*. Note: Visceral granulomatous nodules visualized in Panel B were confirmed to be completely negative for acid-fast bacilli via the modified Acid-Fast cytological staining utilized. (C) Fresh wet-mount peripheral blood smear under phase-contrast microscopy showing massive systemic septicaemia, with dense populations of rod-shaped non-motile bacteria later presumptively identified as *Photobacterium damselae* subsp. *piscicida* circulating between nucleated piscine erythrocytes. (D) Splenic imprint cytology preparation stained via Gram-Color modified kit, revealing dense masses of Gram-negative, bipolar-staining rod-shaped bacterial cells amidst cellular debris later presumptively identified as *Photobacterium damselae* subsp. *piscicida.* (E) Encephalic organ imprint preparation from brain tissue stained via Gram-Color modified kit, demonstrating numerous Gram-negative, rod-shaped bacteria later presumptively identified as *Vibrio* (*Listonella*) *anguillarum* harvested directly from the cerebral microvasculature of fish displaying a characteristic “S” body posture. (F) Fresh wet-mount smear from a cutaneous skin lesion examined under phase contrast microscopy, revealing dense masses of elongated, non-motile filamentous rod-shaped bacteria characteristic of *Tenacibaculum maritimum*.

To differentiate the macro-pathological transformations from potential piscine mycobacteriosis, duplicate touch imprints from all specimens exhibiting visible chronic visceral nodular presentations in the spleen and kidneys (such as those visualized in Fig 2B) were subjected to modified Acid-Fast screening. Microscopic evaluation at 1000 × magnification verified that all of these evaluated nodular tissue samples were completely negative for the presence of acid-fast bacilli.

### Gross pathology and clinical observations

#### *Photobacterium damselae* subsp. *piscicida.*

*P. damselae* subsp. *piscicida* was isolated from fish belonging to all age classes. Affected fish have usually dark body coloration and swim lethargically near the cage surface refusing to feed. According to the water temperature the disease can progress very fast and give significant mortalities (up to 90% especially in younger fish) if oral antibiotic administration is not commenced timely. Spleen, as a rule of thumb appears enlarged with whitish nodules (Fig 2B) and bacteria can be easily detected on stained spleen (Fig 2D) and kidney smears. Often fluid is present in the abdominal cavity. In smaller fish (<50 g), where the disease can progress very fast, whitish nodules might not be visible on the spleen and the bacteria can be found circulating in the blood (Fig 2C). Erratic swimming (frequent swimming bursts, whirling swimming, body “locked” in characteristic “S” position) can be also observed macroscopically and is frequently related to the bacteria present in the brain.

#### Tenacibaculum maritimum.

*T. maritimum* was also isolated from fish belonging to all age classes (2.5−2,500 g) although frequency of occurrence in bigger average weight fish tended to be related with cages management operations that resulted in mechanical damage of the fish. The disease is progressing slowly and in general mortalities can remain controlled if the causative agent is identified and antibiotic administration timely commenced. Macroscopically affected fish appear with pale skin zones which have characteristic yellow edges (Fig 2A) that if left untreated are leading to necrotic lesions. Often, eroded mouth, fins (caudal most often) and gills were detected. Internal organs appeared pale and depending on the severity of the disease fish might be lethargic and refuse to feed. Microscopic examination of wet mounts from material obtained from the superficial wounds reveals presence of elongated, non-motile, filamentous bacteria typical of *T. maritimum* (Fig 2F).

#### Vibrio (Listonella) anguillarum.

*V. anguillarum* was isolated only from fish that had not yet been injection vaccinated (approx. < 15 g) or from fish that had been in the offshore cages system more than 1.5 years (>750 g). Affected fish had dark coloration, pale gills and hemorrhagic areas near the mouth and on the base of fins (Fig 2A). Exophthalmia was detected only on the bigger examined fish while ascites and yellowish-bloody fluid in the intestine was detected in almost all affected examined individuals. As in pasteurellosis, erratic swimming (frequent swimming bursts followed by a resting period during which the fish were sinking, whirling swimming, body “locked” in characteristic “S” position) was detected and was frequently related to the bacteria present in the brain (Fig 2E). Bacteria presence was detected in the spleen, blood (S1 Video), kidneys, brain, and in the rare cases of exophthalmia in eye fluid aseptically withdrawn following surface sterilization of the area.

#### Vibrio harveyi.

Infected fish became lethargic and refused to accept feed and bore clear signs of vibriosis such as external hemorrhages in the base of the fins and mouth and inflammation of the vent. Erratic swimming was often observed especially in bigger fish (>500 g). Hemorrhages were detected in the intestine wall and were more pronounced on the terminal part of it. Ophthalmic lesions were detected only in the bigger fish examined while encephalic congestion was detected on all examined individuals. Bacteria presence was detected in the spleen, blood, kidneys and brain.

#### Vibrio ordalii.

Also, in this case, infected fish became lethargic and ceased feeding. Contrary to the fish affected with *V. anguillarum* and *V. harveyi* erratic swimming, exophthalmia or ophthalmic lesions were not observed and no hemorrhages were detected in the intestinal walls. Bacteria presence was detected in the spleen, blood and kidneys.

#### Vibrio alginolyticus.

Infected fish appeared to be lethargic and dark colored. Ulcers were present on the body surface and scales loosened and were easily lost. Ascites and yellowish-bloody fluid in the intestine was detected in almost all affected examined individuals. Presence of hemorrhages were easily detected on the intestinal wall of the peritoneum and the swimbladder while bacteria presence was easily detected in the spleen, kidneys and blood. The pathogen was recovered from fish belonging to different average weight classes (120−1,800 g) and its presence seemed to be related to intense stress and/or poor growing site conditions (low water exchange, fish subjected to stress following net replacement operations which caused mechanical damage to the skin etc).

### Diagnostic frequency profiles

Descriptive metrics were compiled to map the overall recovery distribution rates of bacterial species and track cross-classified percentage differences (Δ) between pathogen pairs across both analytical layers. The total number and relative proportions of primary mortality drivers at the population scale (*N* = 748 outbreaks) and recovered bacteria (*n* = 2,490 isolates) aggregated across all geographic regions are presented in Fig 3. At the outbreak event level, etiological species were ranked in descending frequency as follows: *Photobacterium damselae* subsp. *piscicida* (48.80%; *N* = 365), *Tenacibaculum maritimum* (35.83%; *N* = 268), *Vibrio* (*Listonella*) *anguillarum* (9.49%; *N* = 71), *V. harveyi* (3.74%; *N* = 28), *V. ordalii* (1.20%; *N* = 9), and *V. alginolyticus* (0.94%; *N* = 7). This outbreak event-level hierarchy directly mirrored the overall isolation frequency, where species were ranked as: *P. damselae* subsp. *piscicida* (46.18%; *n* = 1,150), *T. maritimum* (34.10%; *n* = 849), *V.* (*Listonella*) *anguillarum* (11.41%; *n =* 284), *V. harveyi* (5.26%; *n =* 131), *V. ordalii* (1.77%; *n* = 44), and *V. alginolyticus* (1.29%; *n* = 32).

**Fig 3:**
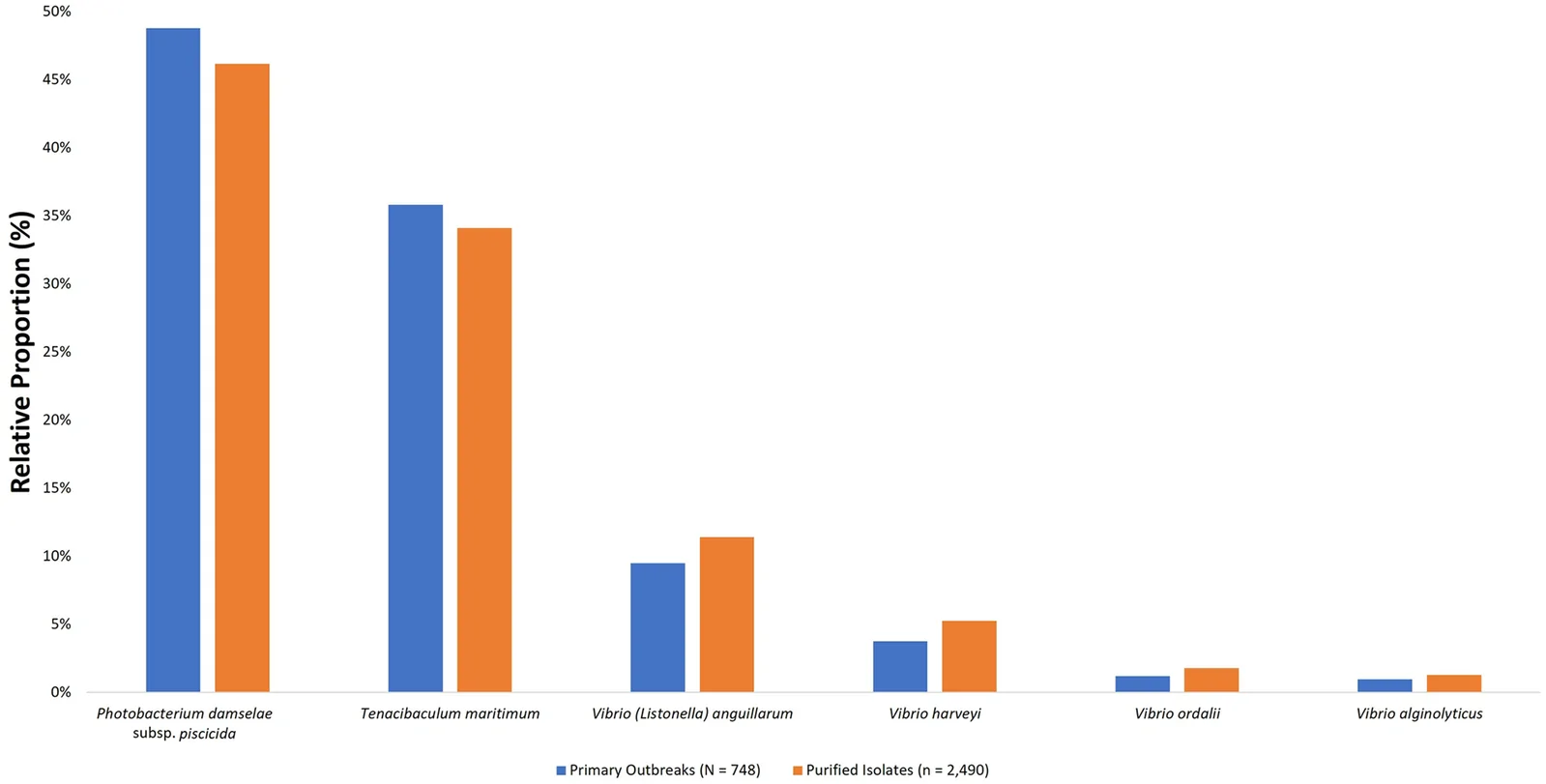
Overall proportional distributions of primary bacterial etiological drivers per outbreak event (*N* = 748) and subsequent laboratory isolation rates (*n* = 2,490) across the multi-year surveillance window. Species-specific metrics reflect individual fish-level recoveries following an event-driven convenience sampling protocol of 2 to 7 highly symptomatic fish per active cage crisis. Pathogen identifications are presumptive based on paired macro-pathology, blood and organ cytology, and phenotypic biochemical profiling.

To describe regional pathogen dynamics, baseline etiological frequencies were evaluated hierarchically across both nested data layers. At both the population outbreak event scale (*N* = 748 distinct cage crises) and the localized laboratory culture scale (*n* = 2,490 purified bacterial isolates), *P. damselae* subsp. *piscicida* and *T. maritimum* consistently emerged as the predominant etiological agents across the coastline, outcompeting the remaining four Vibrionaceae species within the convenience surveillance framework.

These descriptive metrics highlight the sharp frequency differentials separating the highly dominant pathogens (*P. damselae* subsp. *piscicida* and *T. maritimum*) from the four remaining *Vibrionaceae* species along the coastline. The explicit matrix tracking these cross-level percentage differences is detailed in Fig 4.

**Fig 4:**
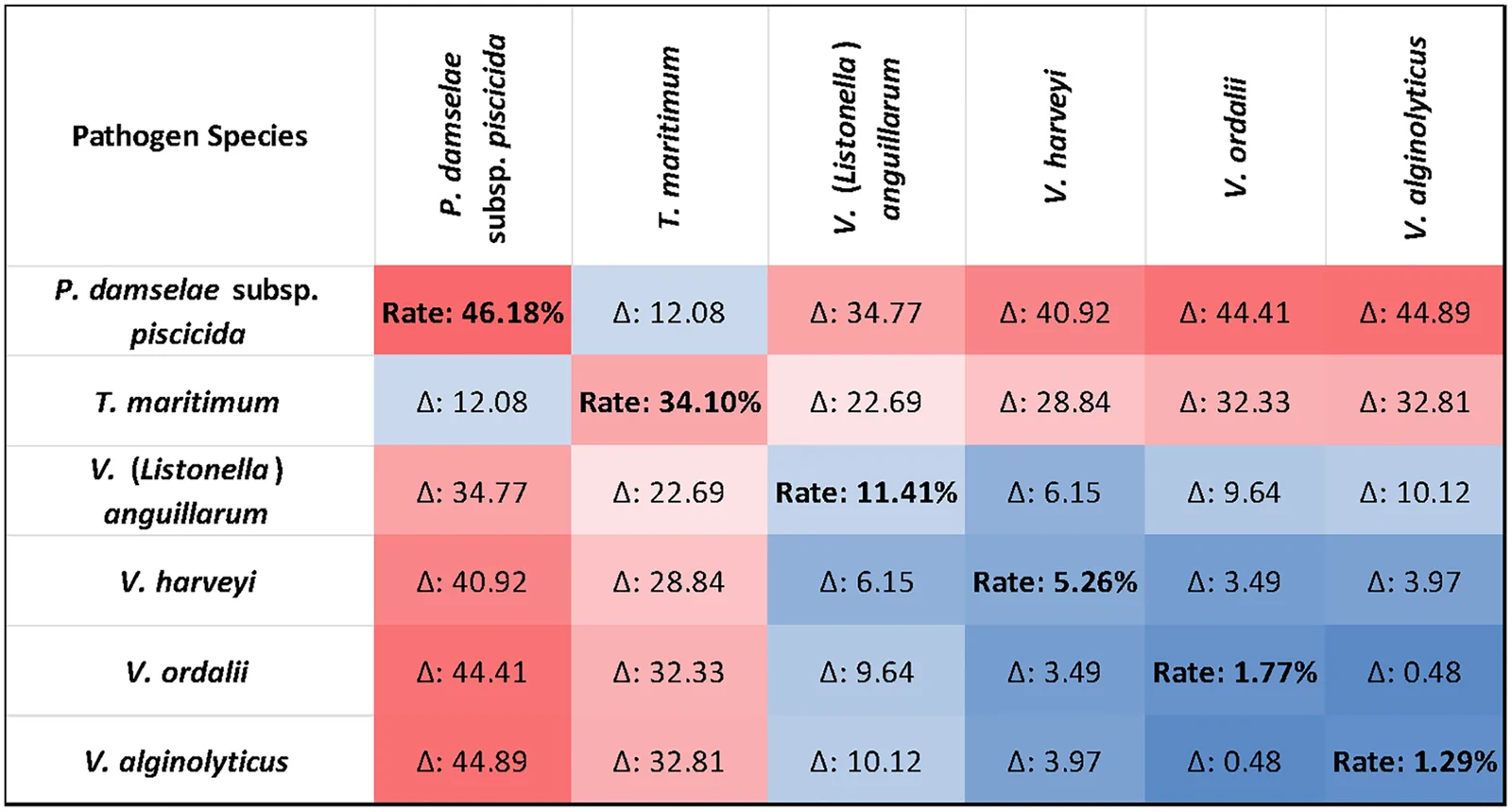
Matrix illustrating the descriptive cross-classified percentage differences between isolated bacterial species. The matrix diagonal displays the raw baseline proportions within the primary isolate pool (*n* = 2,490), while the off-diagonal delta values (Δ) signify the absolute percentage distribution variations between pathogen pairs to illustrate etiological hierarchy density without inferential constraints.

The percentage distribution profiles of bacterial pathogens partitioned by macro-regional surveillance zones are shown in Fig 5. Descriptive comparisons of the relative pathogen distribution frequencies across the three sampling sectors reveal localized fluctuations in isolation rates. These percentage distribution profiles track background variations across the surveyed coastal zones without inferential assertions of population-wide regional equivalence. Despite minor localized shifts in specific secondary pathogen categories, the raw numerical distributions demonstrate that the descriptive baseline pressure remains uniformly anchored by *P. damselae* subsp. *piscicida* and *T. maritimum* across the collective mariculture network.

**Fig 5:**
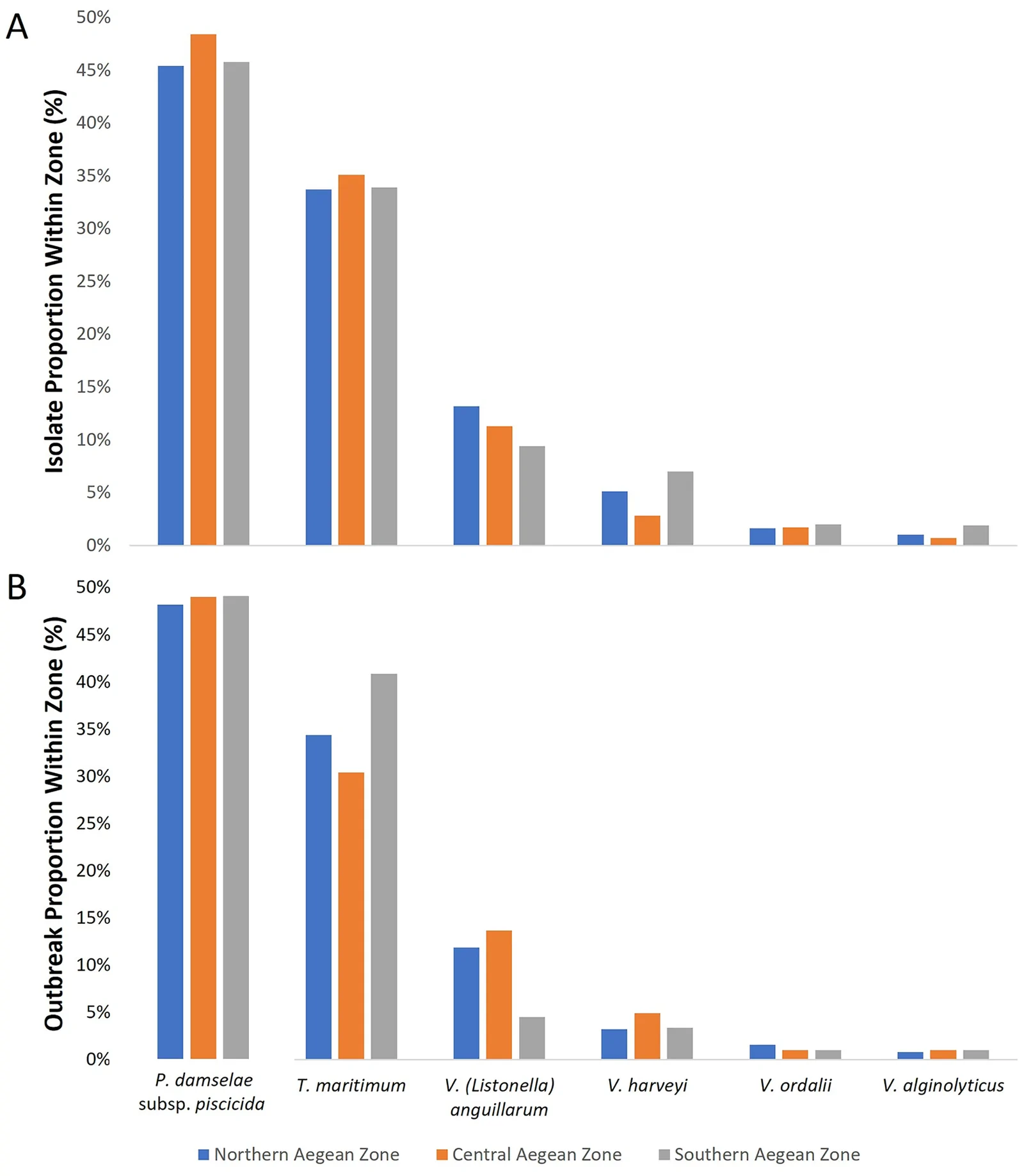
Macro-regional epidemiological variations of bacterial pathogens along the Aegean coast of Türkiye. (A) Relative distribution frequencies across purified laboratory isolates recovered from positive clinical cases across the Northern (*n* = 1,064), Central (*n* = 541), and Southern (*n* = 885) Aegean Surveillance Zones. (B) Relative proportions of primary etiological drivers across distinct cage-level outbreak events partitioned by the Northern (*N* = 253), Central (*N* = 204), and Southern (*N* = 291) Aegean Surveillance Zones.

## Discussion

This multi-year macro-regional epidemiological survey along the Aegean coast of Türkiye provides a comprehensive baseline of the primary bacterial pathogens impacting commercial European sea bass aquaculture offshore production. The observed pathobiological distribution clearly establishes *Photobacterium damselae* subsp. *piscicida* and *Tenacibaculum maritimum* as the primary dominant etiological drivers of disease outbreak events across the surveyed Aegean operations, outcompeting all evaluated *Vibrio* species. This overwhelming dominance highlights a critical biosecurity challenge. Epidemiologically, the persistent recovery of *P. damselae* subsp. *piscicida* within routinely vaccinated individuals serves as a descriptive field hypothesis, suggesting potential lines of future investigation regarding open-sea exposure duration or the spatial distribution of local wild-type strains along the coastline.

While minor localized fluctuations are visible within individual macro-regional zones, the descriptive dataset demonstrates that the absolute distribution patterns follow a closely shared core bacterial profile along the coastline. This baseline trend indicates that mariculture operations face closely shared baseline bacterial threat profiles across the three surveyed sectors, suggesting that coordinating health monitoring models and biosecurity protocols on a macro-regional scale (Fig 1) could offer logical logistical advantages over fragmented, facility-specific strategies.

### *Photobacterium damselae* subsp. *piscicida*

*P. damselae* subsp. *piscicida* remains the primary dominant etiological driver of clinical outbreaks across the collective dataset, continuing to cause significant economic losses in commercial marine aquaculture. In this study, this species was consistently recovered across all three macro-regional surveillance zones, acting as the primary causative driver behind 48.80% of population-scale cage outbreaks (*N* = 365) while concurrently representing the most abundant pathogen within the total isolate pool (46.18%; *n* = 1,150; Table 1). While several monitored mariculture operations incorporate commercial vaccination practices against pasteurellosis, the field observation of recurring infections serves as a realistic descriptive baseline hypothesis for future investigation. These descriptive tracking patterns may reflect the unique challenges of protecting farmed populations across an extended open-sea grow-out duration, or point to a high diversity of wild-type strains naturally circulating within the regional marine environment [7]. Given that hyperacute mortalities can rapidly expand in juvenile fish during environmental shifts [7,8], these descriptive field patterns underline the necessity for future controlled laboratory trials. Systematic evaluations focusing on specific virulence profiles, outer membrane proteins, and antigen configurations of regional wild-type strains are required to validate these field observations and optimize future vaccine formulation designs.

While pasteurellosis outbreaks traditionally manifest in seawater temperatures ranging from 19°C to 23°C [8], the present investigation confirms that clinical disease can occur at lower environmental thresholds. *P. damselae* subsp. *piscicida* was successfully isolated from moribund fish in seawater temperatures as low as 17°C, though associated mortalities remained relatively contained [12,19]. This cold-water recovery supports previous observations indicating that the pathogen has become firmly endemic within the sea bass mariculture networks of the Aegean Sea, expanding its active epidemiological windows beyond classic high-temperature seasonal peaks described in the literature [12].

The manifestation of widespread whitish necrotic nodules throughout the splenic and renal parenchyma in advanced field outbreaks represents a significant diagnostic challenge, as these chronic lesions macroscopically mimic the nodular pathomorphology of piscine *mycobacteriosis* [8]. While definitive exclusion of slow-growing Mycobacterium species requires specialized culture, histopathology, or molecular testing, we deployed a targeted microscopic exclusion strategy via duplicate tissue imprints. Through this cytological approach, acid-fast bacilli were not detected by the method used, indicating that the recorded visceral granulomatous configurations appeared highly characteristic of chronic presumptive pasteurellosis presentations (Fig 2B, 2D) [7,8].

### Tenacibaculum maritimum

In modern intensive mariculture, *T. maritimum* functions as a significant primary mucosal pathogen capable of disrupting open-sea operations [11,20], even when therapeutic interventions mitigate acute losses. In this study, *T. maritimum* was consistently recovered across the three designated macro-regional surveillance zones, acting as the primary causative driver behind 35.83% of population-scale cage outbreaks (*N* = 268) while representing the second most frequent pathogen within the total isolate pool (34.10%; *n* = 849; Table 1).

While *T. maritimum* was highly abundant across field clinical cases, an apparent technical discrepancy was noted in its quantitative recovery rates relative to *P. damselae* subsp. *piscicida* (Table 1) [21,22]. This variance stems from two distinct factors: structural logistical challenges during prolonged field sampling campaigns—where specialized Marine Agar 2216 was not universally available at the immediate point of necropsy—and the clinical reality of field diagnostics. In many acute diagnostic scenarios, the characteristic filamentous morphology of *T. maritimum* can be reliably verified via rapid microscopic examination of fresh wet-mount smears from cutaneous and gill lesions (Fig 2F), rendering subsequent standard bacterial isolation secondary to immediate biosecurity and health management interventions [23,24].

### *Vibrio* (*Listonella*) *anguillarum*

*V.* (*Listonella*) *anguillarum* was successfully isolated across all three geographic macro-regions, acting as the primary causative driver behind 9.49% of population-scale cage outbreaks (*N* = 71) while representing the third most frequently recovered pathogen within the baseline dataset at the individual isolate level (11.41%; *n =* 284; Table 1). Distinctly, the epidemiological distribution of this pathogen exhibited a clear bimodal pattern that targeted two specific fish size thresholds: small, newly transferred juveniles under 15 g and large, market-size fish exceeding 750 g. The occurrence of these infections within these specific size groups serves as an untested descriptive field hypothesis regarding size-dependent pathogen exposure risks along the coastline, since precise population-wide demographic denominators were contractually restricted [10,25–27].

The manifestation of clinical vibriosis in juveniles may indicate localized biosecurity variations in land-based facilities or immediate exposure to elevated wild-type environmental pathogen pressures before standard open-sea transfer. Conversely, the re-emergence of acute infections in large adult fish that have been held in offshore cages for more than 14–24 months represents a realistic field hypothesis concerning the challenges of managing fish across an extended open-sea grow-out duration. For example, some of the diseased adult fish evaluated during this multi-year window were more than 32 months old, whereas during a single elevated-temperature year—where seawater temperatures did not drop below 17.2°C—diseased fish at an average weight of 750 g were just 14 months old. These baseline findings suggest that mariculture management frameworks should evaluate extended production feasibilities for long-cycle market fish. However, since these trends remain untested hypotheses within our convenience surveillance framework, controlled laboratory trials and prospective field monitoring are essential to formally validate these operational dynamics. Future research should therefore incorporate systematic serum antibody titrations and targeted cross-protection modeling. Consequently, definitive regional management modifications or absolute vaccine performance assessments cannot be determined from these descriptive data alone [28,29].

### Minor and Opportunistic Vibrionaceae Species

The remaining three Vibrio species—*Vibrio harveyi, V. ordalii*, and *V. alginolyticus*—acted as primary causative drivers behind only 3.74% (*N* = 28), 1.20% (*N* = 9), and 0.94% (*N* = 7) of population-scale cage outbreaks, respectively, while representing the least abundant diagnostic components within the total isolate pool (5.26%, *n = 131;* 1.77%, *n =* 44; and 1.29%, *n* = 32 respectively; Table 1). In agreement with established literature [7,8], these pathogens lacked a distinct emergence trigger [30]. While the low recovery frequencies of these particular *Vibrio* species across the three surveyed macro-regions cannot mathematically demonstrate adequate background environmental conditions or husbandry quality within this event-driven framework, these descriptive baseline profiles show that opportunistic secondary pathogen pressure remained controlled during the registered cage crises, preventing the widespread polymicrobial epizootics typically associated with severe host physiological stress. It is worth noting, however, that *V. harveyi* has emerged as a primary, highly virulent pathogen in the western and central Mediterranean regions [31,32]. While the present data indicate that *V. harveyi* remains a minor secondary factor along the Aegean coast of Türkiye, continuous diagnostic surveillance is required to monitor for potential shifts in wild-type strain virulence dynamics.

The identification of 142 dual-pathogen presentations highlights the complex, multi-factorial pathological dynamics operating within intensive offshore cage systems (Table 1). These cases consistently involved a surface-to-systemic partitioning, which supports two distinct epidemiological hypotheses. First, primary epithelial erosion from tissue-destructive mucosal pathogens—most notably *T. maritimum*—may have breached the physical cutaneous barrier to establish an anatomical entry portal for secondary, systemic invaders, a mechanism consistent with established subcutaneous infection and gateway models [7,20,21]. Alternatively, these dual configurations may reflect the impact of external anthropogenic stressors, such as mechanical grading, netting friction, or sorting operations. These routine handling practices can cause concurrent physical skin damage and acute physiological stress, thereby provoking the systemic proliferation of internal opportunistic pathogens while facilitating the rapid colonization of superficial cutaneous lesions by *T*. *maritimum* [21,24]. These multi-species interactions suggest that marine field outbreaks are frequently driven by polymicrobial dynamics rather than isolated single-agent models, which must be accounted for when designing broad-spectrum macro-regional biosecurity protocols.

Although recent bibliographic references identify *Aeromonas veronii* as an emerging pathogen in both Greece and Türkiye [33–35], it was not detected or isolated in the present study. This zero-recovery profile, corroborated by the complete absence of characteristic clinical signs of aeromoniasis such as systemic jaundice or distinct yellowing at the fin bases [33,35], serves strictly as an untested field observation within the limits of our diagnostic sensitivity. Thus, this non-recovery cannot definitively establish limited regional spread or absolute absence of the pathogen across the wider geographic basin.

Given the scale of this multi-year surveillance campaign involving 5,235 fish, high-throughput phenotypic profiling was utilized to establish immediate, cost-effective baseline data for regional health management. While whole-genome sequencing of every isolate remains logistically prohibitive for routine industry-scale biosecurity monitoring over a sample size of this magnitude, this practical diagnostic framework provides the immediate, actionable insights necessary for local action plans.

### Study Limitations

Despite its utility in providing broad epidemiological baselines, this field surveillance campaign features clear boundaries that must be contextualized. First, because bacterial characterization relied on phenotypic biochemical profiling keys, species-level taxonomies remain presumptive without universal molecular sequencing, such as 16S rRNA or target PCR, for the entire purified isolate pool. While phenotypic profiling using API 20E system can yield presumptive or variable species-level assignments for specific marine fastidious rods, our diagnostic protocol mitigated these database constraints by strictly pairing biochemical assays with diagnostic organ touch-imprint cytology and characteristic clinical gross lesions. Second, because sampling was event-driven and triggered only when mortality crossed our predefined threshold, the dataset does not represent a randomized longitudinal study and may be subject to localized clustering within individual offshore production sites. Furthermore, because exact husbandry details, vaccine batch histories, and explicit administration timelines were restricted by commercial confidentiality, our documentation of persistent pathogen recovery serves as a realistic descriptive field hypothesis rather than experimental proof of biological vaccine failure. Intermittent field supply variations of Marine Agar 2216 also meant certain *Tenacibaculum* presentations were diagnosed exclusively via cytological microscopy. While these cases were kept strictly within clinical fish metrics to prevent numerical inflation of the purified isolate datasets, this boundary presents a localized ascertainment bias. Consequently, all reported associations with fish size, water temperature, or vaccine status must be interpreted as untested field observations rather than definitive epidemiological correlations.

It must be noted, however, that the event-driven convenience sampling framework employed in this macro-regional campaign was intentionally designed to capture pathogen dynamics during active clinical crises rather than measuring background carrier point-prevalence across healthy individuals. Within an operational aquaculture biosecurity framework, tracking relative isolation frequencies across 748 recorded outbreaks provides a highly realistic, industry-relevant profile of dominant etiological pressures. This focus on active disease states yields actionable baseline data, optimized for regional biosecurity protocols, where identifying the primary drivers of active mortality takes precedence over baseline carrier screening.

## Conclusions

In conclusion, this multi-year surveillance campaign demonstrates that while pasteurellosis (*Photobacterium damselae* subsp. *piscicida*) and tenacibaculosis (*Tenacibaculum maritimum*) remain the dominant etiological drivers of clinical outbreaks, mariculture operations along the Aegean coast of Türkiye face a complex, multi-species bacterial pathogen matrix. The continuous recovery of diverse secondary and opportunistic species highlights that host vulnerability persists across an expansive production window, demanding comprehensive diagnostic coverage. Ultimately, these aggregated field-descriptive findings provide a realistic, industry-wide baseline to shift fish health management from reactive treatments toward predictive, macro-regional biosecurity and screening protocols.

## Supporting information

S1 File Consolidated epidemiological baseline data and pathogen isolation matrices across the three surveillance zones (2020–2025). (XLSX)

S1 Table Centralized list of abbreviations and diagnostic indices used in the manuscript text and biochemical tables. (DOCX)

S1 Video Live motility of presumptively identified *Vibrio* (*Listonella*) *anguillarum* in peripheral blood. Fresh wet-mount preparation of peripheral blood from an infected adult European sea bass (*Dicentrarchus labrax*), recorded via smartphone-mounted ocular microscopy under phase-contrast illumination during routine field diagnostics. The video documents the rapid, darting motility characteristic of rod-shaped bacteria circulating between nucleated piscine erythrocytes, confirming acute systemic septicaemia. Note: Minor frame instability reflects immediate on-site field recording conditions.
(MP4)
