# Supplementary material for: Epidemiological surveillance of bacterial pathogens in cultured European sea bass (Dicentrarchus labrax) along the Aegean coast of Türkiye
Source: PLoS One. 2026 Nov 6;21(11):e0362366. doi: 10.1371/journal.pone.0362366 (PMC13743018; doi:10.1371/journal.pone.0362366)
Supplement: Supplementary file 1 [file pone.0362366.s002.docx]

Table S2. List of abbreviations used in the manuscript text and diagnostic tables.

| Abbreviation | Full Name / Term |
| --- | --- |
| API 20E | Analytical Profile Index 20E |
| BHIA | Brain Heart Infusion Agar |
| TSA | Trypticase Soy Agar |
| TCBS | Thiosulfate Citrate Bile Salts Sucrose |
| MSA-B | Marine Salt Agar with Blood |
| AVMA | American Veterinary Medical Association |
| IACUC | Institutional Animal Care and Use Committee |
| HADYEK | Hayvan Deneyleri Yerel Etik Kurulu |
| ONPG | Ortho-nitrophenyl-beta-D-galactopyranoside |
| ADH | Arginine Dihydrolase |
| LDC | Lysine Decarboxylase |
| ODC | Ornithine Decarboxylase |
| CIT | Citrate Utilization |
| H_2_S | Hydrogen Sulfide Production |
| URE | Urease Activity |
| TDA | Tryptophane Deaminase |
| IND | Indole Production |
| VP | Voges-Proskauer Assay |
| GEL | Gelatinase Liquefaction |
| GLU | Glucose Fermentation |
| MAN | Mannitol Fermentation |
| INO | Inositol Assimilation |
| SOR | Sorbitol Metabolism |
| RHA | Rhamnose Fermentation |
| SAC | Sucrose Fermentation |
| MEL | Melibiose Metabolism |
| AMY | Amygdalin Assimilation |
| ARA | Arabinose Fermentation |
| OX | Cytochrome Oxidase |
| MOB | Bacterial Motility |
| NaCl | Sodium Chloride |
| WOAH | World Organisation for Animal Health |
| WGS | Whole-Genome Sequencing |
